# Transgenic Pearl Millet Male Fertility Restorer Line (ICMP451) and Hybrid (ICMH451) Expressing *Brassica juncea* Nonexpressor of Pathogenesis Related Genes 1 (*BjNPR1*) Exhibit Resistance to Downy Mildew Disease

**DOI:** 10.1371/journal.pone.0090839

**Published:** 2014-03-06

**Authors:** Ramadevi Ramineni, Vijayakumar Sadumpati, Venkateswara Rao Khareedu, Dashavantha Reddy Vudem

**Affiliations:** Centre for Plant Molecular Biology, Osmania University, Hyderabad, Andhra Pradesh, India; Virginia Tech, United States of America

## Abstract

*Brassica juncea* Nonexpressor of pathogenesis-related genes 1 (*BjNPR1*) has been introduced into pearl millet male fertility restorer line ICMP451 by *Agrobacterium tumefaciens-*mediated genetic transformation. Transgenic pearl millet plants were regenerated from the phosphinothricin-resistant calli obtained after co-cultivation with *A. tumefaciens* strain LBA4404 harbouring Ti plasmid pSB111-*bar*-*BjNPR1*. Molecular analyses confirmed the stable integration and expression of *BjNPR1* in transgenic pearl millet lines. Transgenes *BjNPR1* and *bar* were stably inherited and disclosed co-segregation in subsequent generations in a Mendelian fashion. Transgenic pearl millet hybrid ICMH451-*BjNPR1* was developed by crossing male-sterile line 81A X homozygous transgenic line ICMP451-*BjNPR1*. T_3_ and T_4_ homozygous lines of ICMP451-*BjNPR1* and hybrid ICMH451-*BjNPR1* exhibited resistance to three strains of downy mildew pathogen, while the untransformed ICMP451 and the isogenic hybrid ICMH451 plants were found susceptible. Following infection with *S. graminicola*, differential expression of systemic acquired resistance pathway genes, UDP-glucose salicylic acid glucosyl transferase and pathogenesis related gene 1 was observed in transgenic ICMP451-*BjNPR1* and untransformed plants indicating the activation of systemic acquired resistance pathway contributing to the transgene-mediated resistance against downy mildew. The transgenic pearl millet expressing *BjNPR1* showed resistance to multiple strains of *S. graminicola* and, as such, seems promising for the development of durable downy mildew resistant hybrids.

## Introduction

Pearl millet (*Pennisetum glaucum* [L.] R. Br.) is the fifth most important cereal in the world [1] and the fourth most important cereal crop grown in South Asia in terms of area cultivated. It serves as a staple food for millions of people of semi-arid tropics. In the context of climate change, the crop has great potential as it is tolerant to high temperatures and drought stress [2]. Pearl millet has 14 to 20% protein and its amino acid profile is superior to that of maize and sorghum and is comparable to rice and wheat with a favourable leucine/isoleucine ratio [3], [4]. As compared to maize, wheat and sorghum, the energy density of pearl millet grain is higher, owing to its higher oil content [5]. Pearl millet grain is free from major anti-nutritional factors and the lower omega-6 to omega-3 fatty acid ratio makes it as a favoured food for human health [6], [7]. It serves as an excellent annual forage crop owing to low hydrocyanic acid content in the leaves and stems [8].

Downy mildew is the major biotic constraint of pearl millet production leading to devastating annual crop losses of 20–40% [9]. The causal pathogen of downy mildew of pearl millet, *Sclerospora graminicola*, is an obligate biotroph which converts the panicles into useless tendrils. The fungus is highly heterothallic and reproduces asexually by means of sporangia that liberate motile zoospores. Millions of spores are produced within a very short period leading to high incidence of natural mutations. Sexual reproduction is through the formation of thick walled oospores which survive in the soil for more than 13 years [10]. Progenies of a single oospore could be classified into several distinct pathotypic groups [11]. The occurrence of high natural variation [12] created by both asexual and sexual means of reproduction help the pathogen in its rapid co-evolution with the highly out-crossing host pearl millet. In India, the cultivation of genetically uniform single cross hybrids of pearl millet has nearly doubled average yields despite a considerable shift to more marginal production environments. But the vulnerability of these hybrids to epidemics of the potentially devastating downy mildew disease lead to severe grain yield losses [13].

The rate of progress achieved in pearl millet using conventional breeding, though significant, is slow due to the fact that the conventional breeding methods are tedious, time consuming and require many years. Successful use of biotechnological approaches like marker assisted selection in breeding for QTLs has facilitated the improvement of pearl millet for downy mildew resistance [14]. Genetic transformation of pearl millet provides direct access to an unlimited gene pool and can be used to create novel variation [15].

Expression of *AtNPR1,* a co-activator of TGA family of bZIP transcription factors, in *Arabidopsis* [16] and in other plants [17]–[23] proved effective in offering increased resistance to pathogens. Expression of homologues/paralogues of *AtNPR1* in rice [24], [25], *MpNPR1* in apple [26], *Bn*NPR1 in *Brassica napus* [27], *BjNPR1* in mung bean [28] and rice [29] also exhibited enhanced resistance to pathogens, suggesting that NPR1-mediated systemic acquired resistance (SAR) mechanism is evolutionarily conserved [23], [30] and hence can be utilized in various crop species for activating SAR.

In view of the above scenario, the present study deals with the genetic enhancement of pearl millet for resistance against downy mildew by expressing *BjNPR1* gene for plausible triggering of endogenous SAR pathway. *BjNPR1* expressing transgenic pearl millet as well as hybrid exhibited resistance against three virulent strains *Sg* 384, *Sg* 445 and *Sg* 492 of *S. graminicola* as compared to untransformed plants.

## Results

### Genetic transformation and production of transgenic pearl millet plants using pSB111-*bar*-*BjNPR1*

**Figure 1:**
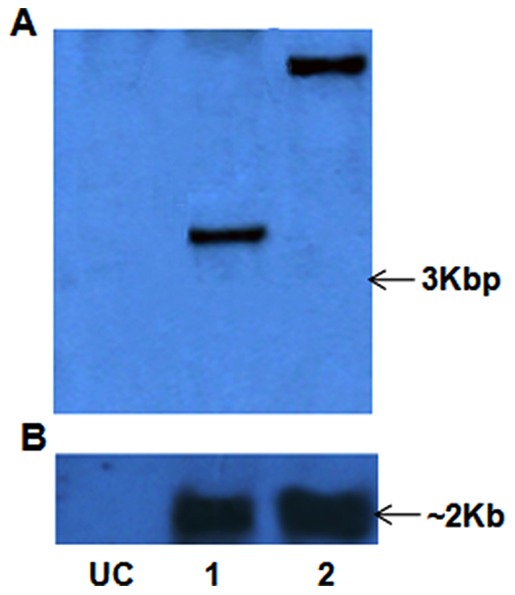
Southern and northern blot analyses of putative ICMP 451- *BjNPR1* transgenics of pearl millet in T_0_ generation. (A) Southern blot of *Bgl*II digested genomic DNA probed with *BjNPR1* sequence showing hybridized bands of >3.0 kbp. (B) Northern blot probed with *BjNPR1* internal sequence showing hybridizable bands of ∼2 kb. Lane UC: Untransformed ICMP 451. Lanes 1&2: Pearl millet *BjNPR1* transgenics 1T_0_ and 2T_0_, respectively.

Two putative transgenic pearl millet ICMP451 plants were regenerated from PPT resistant calli obtained after co-cultivation with the *A. tumefaciens* strain LBA4404 harbouring the super-binary vector (pSB111-*bar*-*BjNPR1*) carrying *bar* and *BjNPR1*. Thirty to forty day-old transformants treated with the herbicide Basta were found to exhibit variable levels of tolerance to the herbicide. PCR analysis of genomic DNA from putative transgenics showed amplified bands of 536 bp and 550 bp corresponding to *bar* and an internal sequence of *BjNPR1* genes, respectively, while untransformed plants failed to show any amplification (data not shown). Southern blot analysis of *Bg1*II digested genomic DNA, probed with 550 bp internal sequence of *BjNPR1* coding sequence, revealed single band of >3 kb (Figure 1A). However, no such band was observed in the untransformed plants under identical conditions. Northern blot analysis of the total RNA probed with internal sequence of *BjNPR1* revealed presence of ∼2 kb *BjNPR1* transcripts only in transgenics (Figure 1B). Both transformants were found fertile and exhibited normal phenotype.

### Inheritance of *bar* and *BjNPR1* genes in T_1_ generation

**Figure 2:**
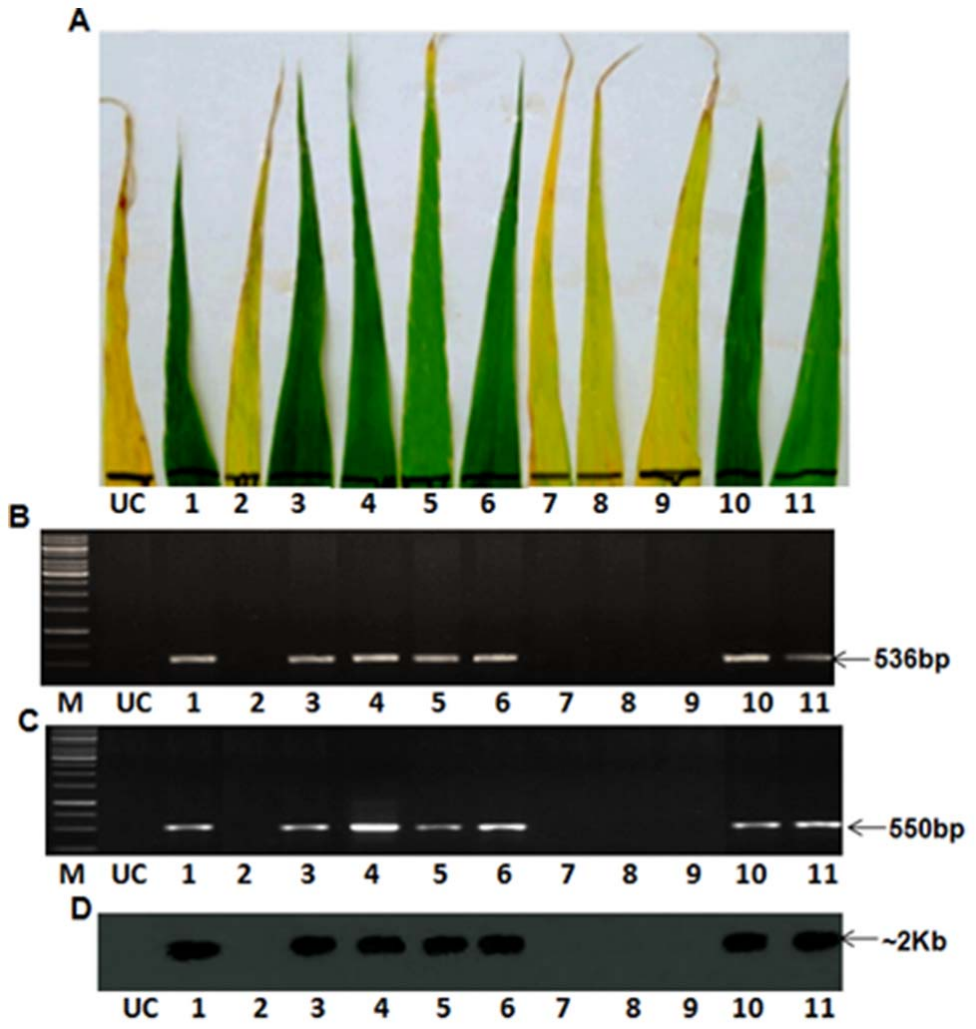
Randomly selected 2T_1_ plants of ICMP 451-*BjNPR1* showing co-segregation of Basta tolerance, amplification of *bar* and *BjNPR1* genes, and expression of *BjNPR1* transcripts. (A) Basta leaf dip assay showing segregation for Basta tolerance and susceptibility. (B) PCR analysis showing segregation for *bar* gene. (C) PCR analysis showing segregation for *BjNPR1* gene. (D) Northern blot showing segregation for expression of *BjNPR1*. Lane M: 1 kb DNA molecular weight marker. Lane UC: Untransformed ICMP 451. Lanes 1 to 11: Randomly selected T_1_ plants of 2T_1_ *BjNPR1*transgenics.

To investigate the inheritance pattern of transgenes, randomly sampled seeds from self pollinated primary transformant (2T_0_) showing better expression of transgene, were germinated and T_1_ plants were grown to maturity in the glass house. Basta leaf dip analysis of the 2T_1_ *BjNPR1* transgenic plants segregated into 19 Basta-tolerant and 6 Basta- susceptible plants giving a ratio of 3:1. Eleven randomly selected T_1_ plants when subjected to PCR analysis, revealed amplification of *bar* and *BjNPR1* sequences in 7 plants while the remaining 4 plants failed to show the amplification of either sequence (Figure 2B & 2C). Seven PCR positive plants also showed tolerance to Basta (Figure 2A) and revealed positive signals in northern blots (Figure 2D) when probed with *BjNPR1* internal sequence. On the other hand, the remaining four plants were susceptible to Basta and failed to show PCR amplification and signals corresponding to *BjNPR1* in northern blot (Figure 2).

### Bioassays of transgenic plants for resistance against *S*. *graminicola*

**Figure 3:**
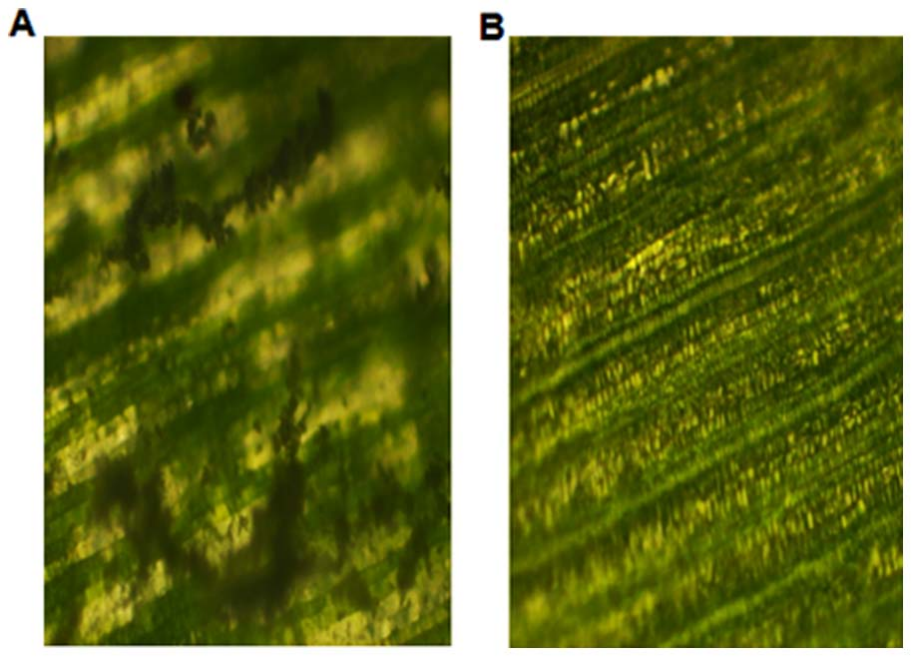
Microscopic view of *S. graminicola* strain *Sg* 384 infected leaf surface of transgenic ICMP451-*BjNPR1* and untransformed ICMP451. (A) Untransformed ICMP451 leaf showing mycelial growth on leaf surface. (B) Transgenic ICMP 451-*BjNPR1* leaf surface showing healthy growth without any mycelia.

In T_2_ generation, out of 17 progenies challenged with three virulent strains of *S. graminicola*, 4 progenies, viz., 2–3T_2_, 2–6T_2_, 2–9T_2_ and 2–12T_2_ showed no segregation for disease susceptibility. Four progenies (2–4T_2_, 2–14T_2_, 2–15T_2_ and 2–16T_2_) were found susceptible, while the remaining 9 progenies showed segregation for 3 resistant plants: 1 susceptible plant (Table 1). Microscopic examination of infected leaf surface of ICMP451 showed extensive mycelial growth and tissue damage, while ICMP451-*BjNPR1* infected leaves were found healthy and showed no damage (Figure 3).The seedlings of untransformed ICMP451 revealed extensive disease symptoms, leading to stunted growth and seedling death. Based on the response to downy mildew reaction, the 17 progenies conformed to the ratio of 1 homozygous resistant: 2 segregating hemizygotes: 1 azygous susceptible. The homozygous lines of *BjNPR1*-transgenics in T_2_ generation showed consistent resistance response to infections caused by three virulent strains.

**Table 1:** Fungal bioassays for downy mildew resistance in T_2_ generation plants challenged with *S. graminicola* strains.

Plant Progeny	Strain *Sg* 384			Strain *Sg* 445			Strain *Sg* 492		
	Resistant Plants (R)	Susceptible Plants (S)	Chi square	Resistant Plants (R)	Susceptible Plants (S)	Chi square	Resistant Plants (R)	Susceptible Plants (S)	Chi square
2–1T_2_ *BjNPR1* ^**^	38	16	0.62	47	16	0.005	42	16	0.207
2–2T_2_ *BjNPR1* ^**^	69	19	0.55	66	25	0.297	68	28	0.889
2–3T_2_ *BjNPR1* *	26	0	-	31	0	-	34	0	-
2–4T_2_ *BjNPR1* ^***^	0	18	-	2	35	-	2	24	-
2–5T_2_ *BjNPR1* ^**^	89	18	3.816	81	18	2.45	60	19	0.038
2–6T_2_ *BjNPR1* *	32	0	-	43	0	-	35	0	-
2–7T_2_ *BjNPR1* ^**^	84	25	0.248	67	24	0.092	73	23	0.056
2–8T_2_ *BjNPR1* ^**^	58	20	0.017	60	24	0.57	54	21	0.36
2–9T_2_ *BjNPR1* *	64	2	-	65	0	-	86	1	-
2–10T_2_ *BjNPR1* ^**^	77	23	0.213	78	26	0	70	24	0.014
2–11T_2_ *BjNPR1* ^**^	49	12	0.923	45	10	1.36	28	9	0.01
2–12T_2_ *BjNPR1* *	87	3	-	95	0	-	83	2	-
2–13T_2_ *BjNPR1* ^**^	43	17	0.356	63	20	0.04	45	15	0
2–14T_2_ *BjNPR1* ^***^	1	20	-	1	13	-	2	24	-
2–15T_2_ *BjNPR1* ^***^	0	14	-	0	9	-	0	12	-
2–16T_2_ *BjNPR1* ^***^	0	17	-	0	24	-	1	22	1
2–17T_2_ *BjNPR1* ^**^	19	7	0.05	17	7	0.22	23	7	0.044
UntransformedICMP451	0	66	-	2	63	-	3	48	-

* Homozygous resistant; ** Heterozygote segregating in a ratio of 3 resistant: 1 susceptible plants; *** Azygous susceptible.

**Figure 4:**
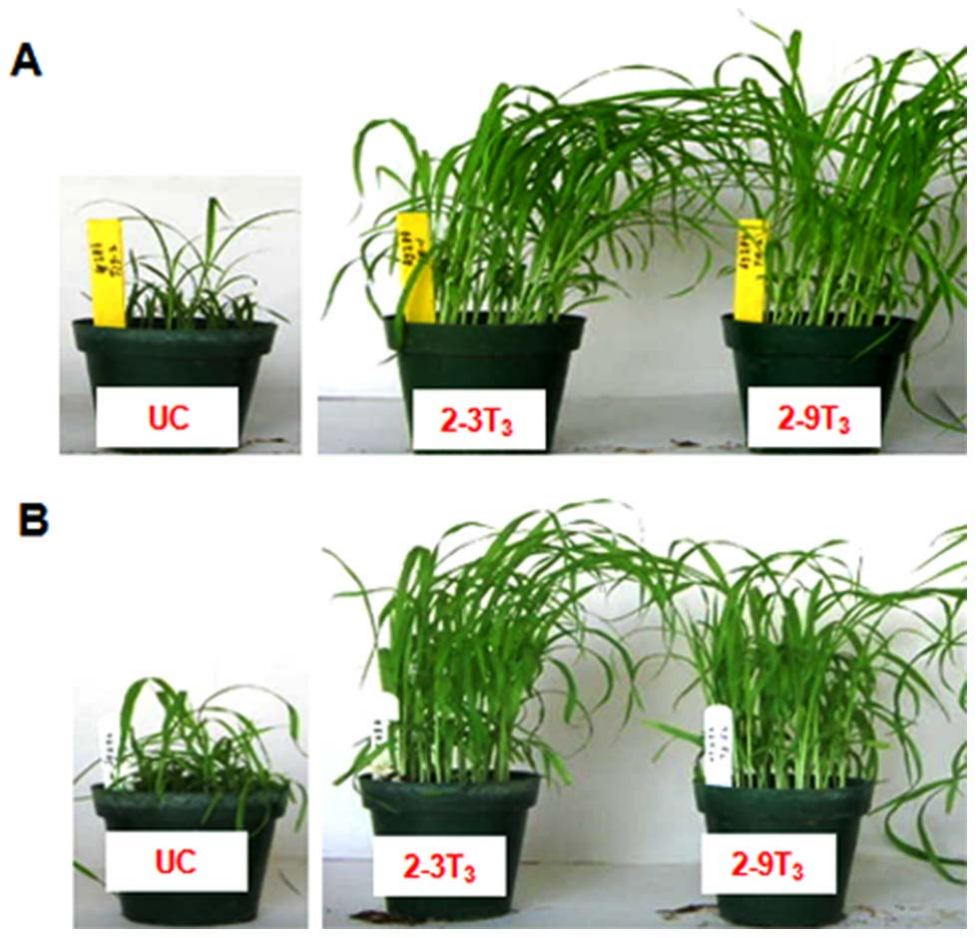
Fungal bioassays on 2–3T_3_ and 2–9T_3_ homozygous ICMP 451-*BjNPR1-*transgenic lines challenged with *Sg* 384 and *Sg* 492 strains of *S. graminicola*. (A) Plants challenged with *Sg* 384. (B) Plants challenged with *Sg* 492 2–3T_3_ and 2–9T_3_: Progenies of ICMP 451-*BjNPR1* exhibiting resistance with healthy and normal growth. UC: Untransformed ICMP 451 exhibiting susceptibility with stunted growth.

**Figure 5:**
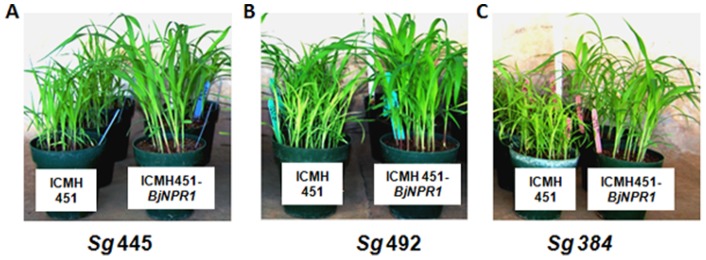
Fungal bioassays on isogenic hybrids ICMH451 (81A x untransformed ICMP 451) and ICMH451-*BjNPR1* (81A x ICMP 451- *BjNPR1*) challenged with *S. graminicola* strains *Sg* 445, *Sg* 492 and *Sg* 384. ICMH 451-*BjNPR1* plants exhibiting resistance with healthy and normal growth. ICMH 451 plants exhibiting susceptibility with stunted growth and chlorotic symptoms.

In T_3_ generation, two resistant homozygous lines 2–3T_3_ and 2–9T_3_ of ICMP451-*BjNPR1*, when challenged with *S. graminicola* strains *Sg* 384 and *Sg* 492, disclosed high-level resistance to the disease (Figure 4). Hybrid ICMH451-*BjNPR1*, challenged with three virulent strains of *S. graminicola,* exhibited high-level resistance with more than 91% resistant plants as compared to the isogenic hybrid ICMH451 which showed an average of >87% infected plants (Figure 5; Table 2). The level of disease resistance exhibited by the hybrid ICMH451-*BjNPR1* was found comparable to that of transgenic parent ICMP451-*BjNPR1*.

**Table 2:** Fungal bioassays for downy mildew resistance in isogenic hybrids ICMH451 and ICMH451-*BjNPR1.*

Genotype	Strain *Sg* 384			Strain *Sg* 445			Strain *Sg* 492		
	Plants infected	Resistant Plants	% Resistant plants	Plants infected	Resistant Plants	% Resistant plants	Plants infected	Resistant Plants	% Resistant plants
Untransformed ICMP 451	73	1	1.37	90	6	6.67	71	3	4.25
81A (Male sterile line)	67	2	2.98	110	6	5.45	87	9	10.34
ICMH451-*BjNPR1*	78	72	92.31	108	100	92.59	101	92	91.09
ICMH 451	94	12	12.76	82	10	12.19	101	13	12.87
ICMP451-*BjNPR1*	88	84	95.45	82	77	93.90	87	81	93.10

### Expression of UDP-glucose salicylic acid glucosyl transferase gene (*SAGT*) and Pathogenesis related gene 1(*PR1*) transcripts

**Figure 6:**
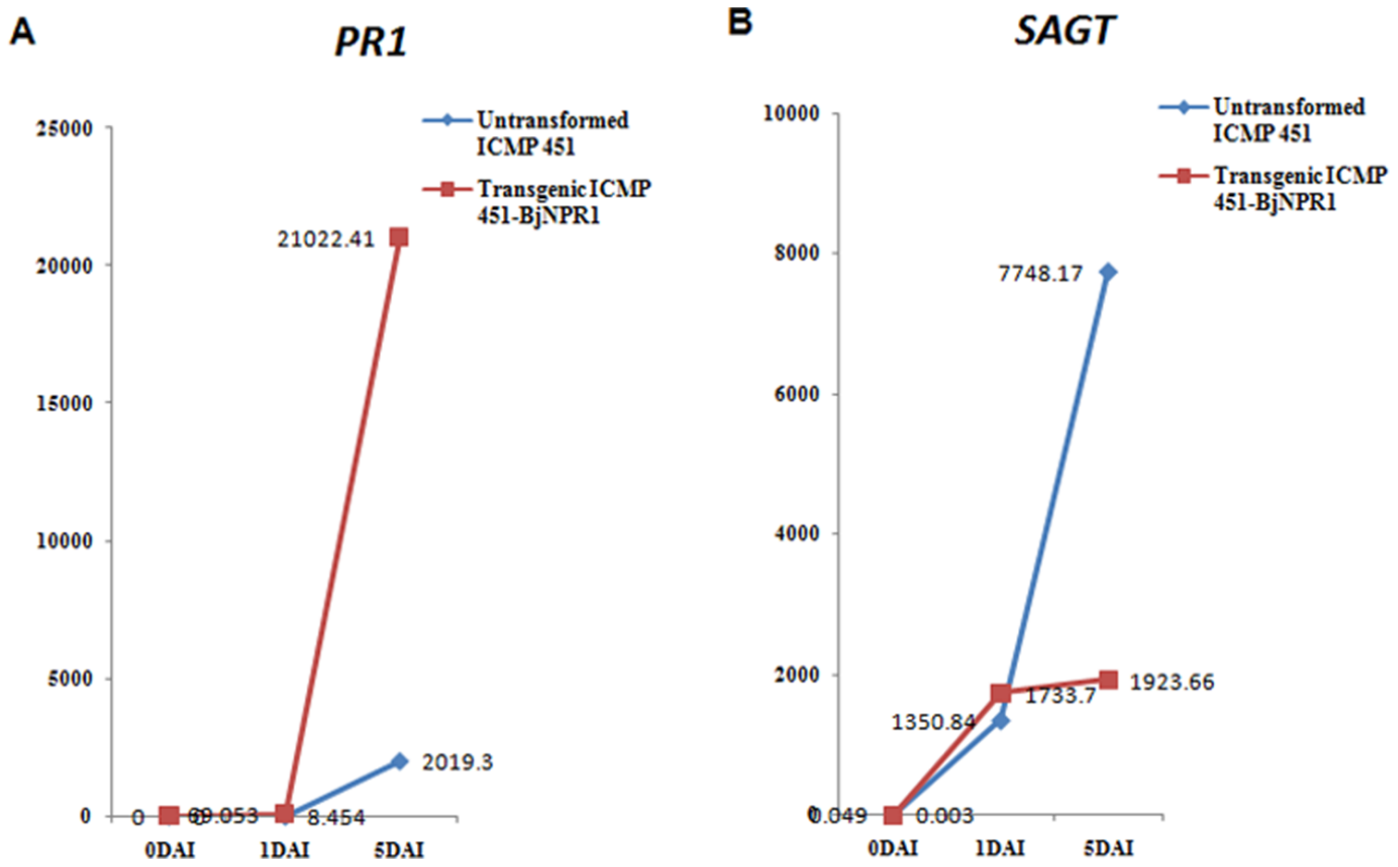
*PR1* and *SAGT* transcript levels in the untransformed ICMP 451 and ICMP 451-*BjNPR1* homozygous plants as normalized against *18S rRNA* gene at 0, 1 and 5 DAI (Days After Infection) with *Sg 384* strain of *S. graminicola.* (All values represented on Y axis are to be multiplied with 10^−5^ to get the actual normalized values).

Expression profiles of two endogenous genes of SAR pathway, viz., *SAGT* and *PR1* were analyzed in untransformed ICMP451 and homozygous 2–3T_3_ ICMP451-*BjNPR1* transgenic plants challenged with *S. graminicola* strain *Sg* 384. *SAGT* transcripts revealed basal level expression in uninfected untransformed (0.049) and transgenic (0.003) plants, while no transcripts of *PR1* could be detected. Transcript levels of *PR1* in the infected transgenic plants were elevated by 8.16 and 10.41 times as compared to infected untransformed plants at 1 and 5 days, respectively, after infection (Figure 6A and Table 3). After one day of infection, a slight increase of 1.28- fold in the *SAGT* transcript levels was recorded in the infected transgenic plants compared to infected untransformed plants. However, after fifth day of infection, infected untransformed plants showed a 4-fold increase in the *SAGT* transcripts as compared to the infected transgenic plants (Figure 6B and Table 3).

**Table 3:** Normalized transcript values of *PR1* and *SAGT* in the Untransformed ICMP 451 and transgenic ICMP 451- *BjNPR1* challenged with *S. graminicola* strain, *Sg* 384.

	Gene	Untransformed ICMP 451	Transgenic ICMP 451- *BjNPR1*
**Before Infection**	*PR1*	*-*	-
	*SAGT*	0.049×10^−5^	0.003×10^−5^
**1Day after infection**	*PR1*	8.454×10^−5^	69.053×10^−5^
	*SAGT*	1350.84×10^−5^	1733.70×10^−5^
**5Days after infection**	*PR1*	2019.30×10^−5^	21022.41×10^−5^
	*SAGT*	7748.17×10^−5^	1923.66×10^−5^

### Sub-cellular localization of *BjNPR1-Gfp* fusion protein in pearl millet

**Figure 7:**
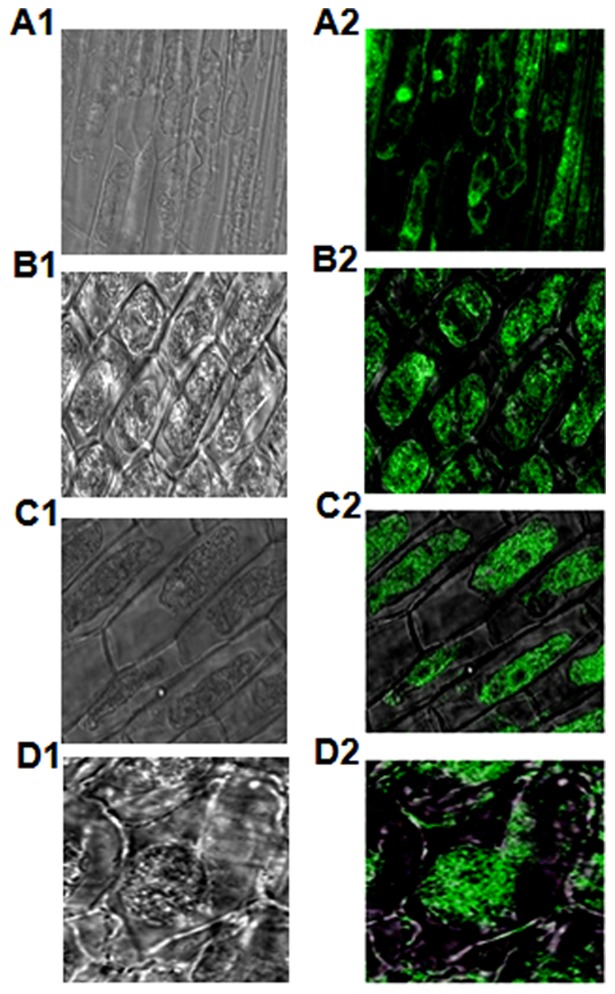
Sub-cellular localization of GFP/ BjNPR1-GFP fusion protein before and after salicylic acid treatment in pearl millet. (A and B) Leaf sheath cells transiently expressing GFP showing green fluorescence both in the cytoplasm and nucleus, before and after salicylic acid treatment, respectively. (A1) Bright field image. (A2) Overlay image. (B1) Bright field image (B2) Overlay image. (C) Leaf sheath cells transiently expressing BjNPR1-GFP showing green fluorescence in the cytoplasm before salicylic acid treatment. (C1) Bright field image. (C2) Overlay image. (D) Leaf sheath cells transiently expressing BjNPR1-GFP showing green fluorescence in the nucleus after salicylic acid treatment. (D1) Bright field image. (D2) Overlay image.

Fluorescence microscopy of pearl millet leaf sheaths electroporated with p*Gfp* revealed localization of GFP protein in the cytoplasm and nucleus both before and after SA treatment. Whereas, the chimeric fusion protein BjNPR1-GFP was found localized only in the cytoplasm prior to salicylic acid treatment and was translocated into the nucleus after salicylic acid treatment (Figure 7).

### Tolerance of *BjNPR1*- transgenic plants to salicylic acid

**Figure 8:**
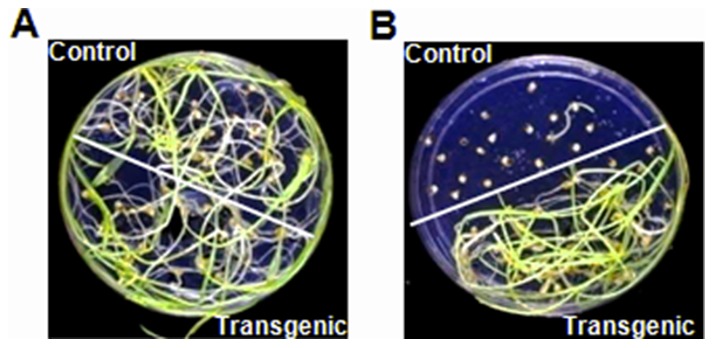
ICMP 451-*BjNPR1* transgenics exhibiting tolerance and untransformed ICMP 451 exhibiting sensitivity to 400µM salicylic acid. (A) Seeds of ICMP 451-*BjNPR1* transgenics and untransformed ICMP 451showing normal germination on MS medium without salicylic acid. (B) Seeds of ICMP 451-*BjNPR1* transgenics showing normal germination on MS medium supplemented with 400 µM salicylic acid, while untransformed ICMP 451 failed to germinate.

Tolerance of *BjNPR1-* transgenic plants to salicylic acid was assessed for seed germination and seedling growth on the MS medium supplemented with different concentrations of salicylic acid. T_4_ homozygous ICMP451 *BjNPR1-*transgenic plants were able to germinate and grow normally on the MS media supplemented with 50 µM to 400 µM salicylic acid, whereas seeds of untransformed ICMP451 failed to germinate on MS medium supplemented with >100 µM salicylic acid (Figure 8).

## Discussion

Pathogens infect a wide range of crops, which have evolved innate and inducible resistance. Induced resistance is often suppressed by pathogens. Such competitive evolution leads to breaking down of resistance. Downy mildew pathogen of pearl millet evolves rapidly resulting in the quick breakdown of resistance. Genetic engineering of disease-resistance through transfer of plant defense-related genes or pathogen-originated genes into crops is valuable in terms of cost, efficacy and reduction of pesticide usage [22]. Engineering for broad spectrum disease resistance against downy mildew in pearl millet is essential for the sustained crop productivity. Employment of transcription factors responsible for the activation of genes involved in SAR may potentially contribute for the development of long lasting resistance against a wide range of rapidly evolving pathotypes [31]. Employing optimized protocols, the co-integrated super-binary vector pSB111-*bar*-*BjNPR1* was used for the development of transgenic pearl millet plants. Putative transgenic ICMP451-*BjNPR1* plants that survived in the glass house showed tolerance to Basta, amplification of *bar* and *BjNPR1* genes, indicating their transformed nature. Southern blot analyses of the *Bg1*II digested genomic DNA of putative transgenics probed with *BjNPR1* have revealed the presence of a single band of >3 kbp (Figure 1A), establishing the single site integrations of transgenes. Northern blot analysis of the primary transformants clearly showed the expression of transgene *BjNPR1* (Figure 1B). It was reported that single copy integration of transgene(s) is essential to achieve predictable patterns of inheritance and to eliminate the problem of gene silencing in the transgenic plants [32]. Segregation analyses of transgenes in T_1_ progenies conformed to the monogenic ratio (3:1) for herbicide tolerance, PCR and northern analyses (Figure 2), testifying that these genes are stably integrated into the pearl millet genome. The co-segregation of transgenes established that *bar* and *BjNPR1* are integrated and manifest at the same site and transmitted together to the next generation is in conformity with earlier reports [33], [34].

The efficiency of *BjNPR1* gene in conferring resistance to downy mildew was evaluated in T_2_ generation by challenging the transgenic plants with three *S. graminicola* strains viz., *Sg* 384, *Sg* 445 and *Sg* 492 [35]. Bioassays on 17 progenies of 2T_1_ ICMP451-*BjNPR1* revealed distinct segregation into 4 homozygous resistant, 9 heterozygous (segregating for 3 resistant: 1 susceptible) and 4 susceptible azygous progenies depicting 1:2:1 monohybrid segregation. Furthermore, in T_3_ generation, two homozygous lines (2–3T_3_ and 2–9T_3_) were screened for resistance against two highly virulent strains *Sg* 384 and *Sg* 492. The progenies of both 2–3T_3_ and 2–9T_3_ displayed superior resistance (97% resistant plants) upon challenging with *Sg* 384 and *Sg* 492. These observations on T_3_ progenies clearly signify the transmission of *BjNPR1* gene into progeny plants and the high degree of disease resistance conveyed by the *BjNPR1* transgene. Similarly, the hybrid ICMH451-*BjNPR1* exhibited high level (>91% plants without infection) resistance as compared to isogenic hybrid ICMH451 (>87% infected plants) when challenged with the three virulent strains of *S. graminicola* (Table 2), suggesting broad spectrum resistance conferred by *BjNPR1* gene which can be exploited successfully for the production of commercial hybrids. Earlier, it was reported that transgenic rice and mungbean expressing *BjNPR1*, manifested enhanced resistance to *Rhizoctonia solani* and *Magnaporthe grisea* pathogens [28], [29]. Similarly, transgenic plants expressing *AtNPR1* disclosed resistance to fungal pathogens, such as *M. oryzae* and *Fusarium verticillioides* in rice [20], [29], to *F. oxysporum*, *Stemphylium solani* and *Ralstonia solanacearum* in tomato [22], to *F. graminearum* in wheat [21], to *Botrytis cinerea*, *Alternaria radicina, Sclerotinia sclerotiorum*, *A. radicin* and *E. heraclei* in carrot [19] and to *Verticillium dahliae*, *F. oxysporum*, *R. solani*, *Alternaria alternata* and *Thielaviopsis basicola* in cotton [17], [36].

Earlier, it was reported that transgenic plants over-expressing NPR1, showed a greater and quicker activation of various *PR* genes in response to pathogen challenge [20], [21], [26], [37]. In the current investigation, the expression profiles of two endogenous genes, *PR1* and *SAGT* were analysed in untransformed ICMP451 and homozygous transgenic ICMP451-*BjNPR1* line (2–3T_3_) infested with *S. graminicola*. Following infection with *S. graminicola*, differential expression of endogenous genes *PR1* and *SAGT* were observed. Absence of *PR1* transcripts before infection in the untransformed ICMP451 and ICMP451-*BjNPR1* transgenic lines may be attributed to the un-induced state of SAR/ absence of SAR signal. Transcript levels of *PR1* in infected ICMP451-*BjNPR1* plants were elevated 8.16 fold and 10.41 fold compared to infected untransformed plants after 1 and 5 days of infection, respectively, thereby contributing to the control of *S. graminicola* infection and imparting transgene mediated disease resistance. *AtNPR1* over-expressing *A. thaliana* plants did not show *PR* gene expression before induction either by chemicals or by pathogen infection [16], [37]. Elevated levels of *PR1* transcripts were observed in *AtNPR1* expressing tobacco plants infested with root-knot nematode [38]. Analysis of *PR1* transcripts in *Brassica juncea,* infected with biotrophic pathogen *Erysiphe cruciferarum*, revealed that the resistance is conferred by sustained, elevated levels of *PR1* transcripts while the susceptible reaction showed low level of *PR1* expression [31].

The transcript levels of *SAGT* in the uninfested conditions are very low both in the control and transgenic plants indicating basal level expression in the absence of infection. An 1.28 fold increase in *SAGT* transcripts in transgenics compared to the untransformed plants at 1day after infection suggest that soon after infection, SAGT levels were slightly elevated by the transgene- *BjNPR1*. *NPR1* mutants of *Arabidopsis* exhibited reduced induction of *AtSAGT1* at 16 h after infection with *Pseudomonas syringae* as compared to the wild-type plants, implying *NPR1* mediated partial up-regulation and immediate early induction of *AtSAGT1* [39]. A four-fold elevated level of *SAGT* transcripts in the infected susceptible untransformed plants as compared to resistant *BjNPR1*-transgenics after 5 days of infection with *S. graminicola* may be attributed to the continued buildup of infection in the untransformed plants while the containment of disease in the transgenics might have resulted in low level expression of *SAGT*. *SAGT* is an established immediate early responsive gene of SA induced SAR [40], [41]. Over-expression of *AtSAGT1* in *Arabidopsis* resulted in reduced levels of both free salicylic acid and its glucosylated forms and increased susceptibility to *P. syringae* [42], implying the importance of down-regulation of *SAGT* after the initial SAR onset. Following fifth day after infection with *S. graminicola*, elevated levels of *PR1* transcripts in resistant *BjNPR1*-transgenics and elevated levels of SAGT transcripts in susceptible untransformed plants, indicate the complex role of NPR1 both in positive and negative regulation of genes in the SAR pathway.

Earlier studies reported that nuclear translocation is essential for *AtNPR1* protein to activate *PR* gene expression [43]. Therefore, in the present study experiments were carried out to confirm the localization of BjNPR1 protein in pearl millet before and after SA treatment by using a transient transformation assay with p*Gfp* 35S/p*BjNPR1-gfp* 35S fusion construct. Fluorescence microscopic observations in leaf sheaths electroporated with p*Gfp* 35S disclosed the localization of GFP protein both in the cytoplasm and nucleus before and after SA treatment. Whereas, the chimeric fusion protein BjNPR1-GFP was localized only in the cytoplasm prior to SA treatment and was translocated to nucleus after SA treatment, suggesting that BjNPR1 protein is capable of nuclear translocation upon activation by elicitors or signal molecules such as SA which mimics the situation of activated defense signaling. Transport of BjNPR1-GFP fusion protein into the nucleus in pearl millet, facilitated by SA treatment, may be attributed to the SA induced changes in redox status of the cytoplasm. Thiol mediated redox changes of the cytoplasm was found to be responsible for the reduced state of AtNPR1 in *Arabidopsis thaliana* [44], [45]. Translocation of a significant amount of BjNPR1-GFP fusion protein into the nucleus was observed in tobacco epidermal layer cells treated with SA [31]. Nuclear localization of NPR1 was found essential for the regulated expression of Isochorismate synthase 1[46], a key enzyme in the SA biosynthetic pathway [47]. Earlier studies reported the translocation of monomeric NPR1 into the nucleus [43] and its interaction with TGA family transcription factors [48]–[50].

T_4_ homozygous ICMP451-*BjNPR1* transgenics were able to germinate and grow normally on MS media supplemented with 50 µM to 400 µM SA, while the seeds of untransformed ICMP451 failed to germinate on MS medium supplemented with >100 µM SA, suggesting that expression of transgene *BjNPR1* confers tolerance to SA in pearl millet. Besides activation of SAR pathway, NPR1 also offers tolerance to SA owing to its homoeostatic regulatory role on genes involved in the SA biosynthesis and its utilization [46], [51]. Reduced levels of free-SA were observed in *AtNPR1* over-expressing rice plants [52] consistent with an active role for AtNPR1 in feedback inhibition of SA accumulation.

The present results represent the first report on the development of downy mildew resistant transgenic pearl millet, deploying a plant regulatory gene, *BjNPR1*. Furthermore, the *BjNPR1* transformants, endowed with high-level resistance, appear promising for commercial cultivation in downy mildew-prone areas besides serving as a novel genetic resource in traditional cross breeding. The BjNPR1 mediated altered expression of endogenous defense genes revealed the multiple ways of regulation of defense responses. As such, BjNPR1 serves as a key factor in SA signal transduction and regulation of genes conferring disease resistance.

## Materials and Methods

### *Agrobacterium tumefaciens-*mediated transformation in pearl millet using super-binary vector pSB111*-bar-BjNPR1*

*A. tumefaciens*-mediated genetic transformation experiments were carried out using LBA4404 strain harbouring pSB111-*bar*-*BjNPR1* super-binary vector [29]. Seeds of pearl millet male fertility restorer line ICMP451 were obtained from the ICRISAT-Patancheru, Hyderabad were used for genetic transformation studies. Mature seeds were surface sterilized with 0.1% (w/v) HgCl_2_ for 10 min followed by three washings with autoclaved distilled water, and were placed on MS basal medium [53] and allowed to germinate in dark at 25±1°C. Later, scutellar regions were cut aseptically and placed on CIM medium (MS medium containing 2 mg/l 2, 4-D, 0.5 mg/l kinetin, tryptophan 50 mg/l and casamino acids 1 g/l) with 0.3% gelrite for callus initiation. After 3 weeks of incubation at 25±1°C under continuous light (3000 lux units), the scutellum-derived calli were used for transformation experiments. *A. tumefaciens* cultures were initiated by inoculating a colony of the bacterium into 6 ml YEP medium containing 50 mg/l spectinomycin and 10 mg/l tetracycline at 225 rpm and 29°C for 20 h. The bacterial culture was pelleted at 3500 rpm and resuspended in 10 ml of PIMII medium [54] supplemented with 100 μM acetosyringone, and incubated for 10 h at 29°C. The cell pellet was collected and re-suspended in 10 ml of CIM liquid medium supplemented with 400 µM acetosyringone and this medium was designated as co-cultivation medium. After 2 h of incubation, the bacterial culture with 1.0 O.D_600_ was used for co-cultivation. Before co-cultivation, the embryogenic calli were cut into small pieces, and were treated with MS basal medium supplemented with 400 µM acetosyringone for 30 min. Later, calli were transferred into the *Agrobacterium* culture (OD_600_ = 1) and left on the shaker at 100 rpm for 30 min. These calli were placed on the co-cultivation medium supplemented with 400 µM acetosyringone and 3.3 mM L-cysteine. Three days after co-cultivation, infected calli were washed thoroughly in MS basal medium supplemented with 100 mg/l cephotaxime and 250 mg/l carbenicillin and allowed to proliferate on CIM medium with 3.3 mM L-cysteine, 100 mg/l cephotaxime and 250 mg/l carbenicillin for one week. Proliferated calli were subjected to three stages of selection of 1 week→10 days→15 days duration on CIM medium with 5, 8 and 12 mg/l Phosphinothricin (PPT), respectively. For regeneration, the actively dividing calli were transferred onto MS medium supplemented with BAP (1.0 mg/l), Kinetin (0.25 mg/l), Sucrose (15 g/l), Sorbitol (15 g/l), Inositol (100 mg/l) and Gelrite (3 g/l) and incubated at 25±1°C under light (3000 lux units). For root development, MS half strength with 2 g/l gelrite was used and the plantlets were transferred to soilrite for acclimatization and then established in the glass house in pots containing soil. Transgenic plants (30–40 day-old) along with untransformed controls were tested for their tolerance to the herbicide Basta [55].

### PCR, Southern and northern blot analyses

Genomic DNA was isolated from the Basta tolerant and untransformed control plants using the method of Zidani et al [56]. PCR analysis was carried out using the primers; 5′-GCC CAT GGA GAC CAT TGC TAG ATT TGA TGA TT- 3′ and 5′ -GCG GAT CCT CAC CGA CGC CGG TGA GAG GGT TTA G – 3′ for *BjNPR1*, and 5′- CTA CCA TGA GCC CAG AAC G – 3′ and 5′- TCA GAT CTC GGT GAC GGG -3′ for *bar-nos*. DNA isolated from the untransformed control plants was used as the negative control and Ti super binary vector was used as the positive control. For Southern blot analysis, 10 μg of genomic DNA was digested with *Bgl*II, electrophoresed on a 0.8% agarose gel and subsequently transferred to an N^+^ Nylon membrane (Amersham Biosciences) and fixed by exposing to UV (1200 μJ for 60 s) in an UV cross linker (Sambrook and Russell, 2001). A 550 bp *BjNPR1* internal sequence was labeled with α-^32^P dCTP employing ready to go random primer DNA labelling kit (Amersham Biosciences). The Southern blots were probed with α-^32^P labeled *BjNPR1* sequence [29]. Similarly, northern blot analysis was carried out using total RNA isolated from the untransformed plants as well as transformants. About 10 μg of RNA was separated on 1.4% denaturing agarose gel and α-^32^P labeled *BjNPR1* sequence was used as probe.

### Development of isogenic Pearl millet hybrids ICMH451and ICMH451-*BjNPR1*

The pearl millet male sterile line 81A (ICMA1) was crossed with homozygous transgenic line (2–3T_3_ ICMP451-*BjNPR1*)/untransformed ICMP451 to produce the isogenic hybrids. The resulting hybrids ICMH451-*BjNPR1* and ICMH451 were evaluated against the downy mildew pathogen.

### Bioassays against downy mildew disease

Bioassays were carried out under standard controlled conditions in a glass house at ICRISAT, Hyderabad, India. Untransformed ICMP451, T_2_ and T_3_ ICMP451-*BjNPR1* transgenics, hybrid ICMH451 and its isogenic hybrid ICMH451-*BjNPR1* plants were evaluated for downy mildew resistance. Seeds were sown in 12 cm diameter pots filled with potting mixture (Alfisol, sand, and farmyard manure in a 2:1:1 ratio) with 1 g Di-ammonium phosphate/kg of soil. The pots were irrigated daily and kept in the greenhouse until inoculation. A day before inoculation, systematically infected leaves was collected from the field and their downy growth is removed with a wet cotton swab. The leaves were cut into small pieces, and placed in humidity chamber and incubated at 20°C and >95% Relative Humidity (RH) in the dark for 6 h to encourage the pathogen to form sporangia. The incubator is programmed to cool down to 5–6°C to prevent the sporangia from germinating. Sporangia produced on the leaves were washed off into cold water (5–6°C). The concentration of sporangia was determined using hematocytometer and adjusted to 1×10^6^ sporangia/ml. Two-day-old potted seedlings at coleoptile to one-leaf stage were inoculated with sporangial suspension of highly virulent strains of *S. graminicola*, *viz*., *Sg* 384, *Sg* 445 and *Sg* 492 with virulence indices of 14.38, 16.46, 12.63, respectively [9] and covered with a moist polythene sheet. These plants were maintained in a chamber at 20±1°C and >95% RH for 12–16 h [57] and then transferred to a greenhouse where the temperature is maintained between 25–30°C for disease development [58]. After 5 days, leaves from both infected transgenic and untransformed plants were visualized under microscope. Based on the disease reaction, the plants exhibiting healthy growth with dark green coloured leaves were classified as resistant; while susceptible plants showed chlorosis initially at the base of the second leaf lamina which progressed to subsequent leaves ultimately leading to stunting and death of the plants. After 14^th^ day of inoculation, the percent of disease incidence was calculated as (number of infected plants/ total number of inoculated plants) ×100 [59]. The plants were grown to maturity under controlled conditions in a glass house and selfed seeds were collected plant-wise for further use.

### Sub-cellular localization of GFP/BjNPR1-GFP fusion protein

*BjNPR1* sequence devoid of stop codon was amplified using 5′- GCGGATCC ATGGAGACCATTGCTAGATTTGATGA-3′ and 5′- GCGGATCCCCGACGCCG GTGAGAGGGTTTAG -3′ primers and ligated at the 5′ end of *gfp* in p*Gfp* 35S plant expression vector [60]. Electroporation of plasmids, p*Gfp* 35S/ p*BjNPR1*-*Gfp* 35S into pearl millet tissue was done independently according to Dekeyser et al. [61]. Leaf base explants from 7 day old, etiolated, pearl millet seedlings were chosen as the explants. The isolated leaf bases were pre-incubated in electroporation buffer (EPR) containing 10% glucose, 4 mM CaCl_2_, 10 mM HEPES, adjusted to pH 7.2. After 3 h of pre-incubation, the explants were incubated for 1 h in a electroporation cuvette containing 250 µl of EPR buffer supplemented with 0.2 mM spermidine and 20 µg of plasmid DNA (p*Gfp* 35S/p*BjNPR1-gfp*). Before electroporation, 11 µl of 3 M NaCl was added to the cuvette and chilled on ice for 10 min. Pulse of 375 V/cm electric field strength was discharged at 900 µF capacitance using a BTX electroporation system. The cuvettes were immediately placed again on ice for 10 min and the explants were rinsed with MS basal medium and incubated on plates containing MS basal medium at 25°C in the dark for 46 h. Fluorescence was visualized using a laser scanning confocal microscope (Leica TCS STED; Leica microsystems, Heidelberg, Germany).

To study the effects of salicylic acid (SA) on sub-cellular localization of BjNPR1-GFP, the electroporated leaf sheaths were incubated at 25°C in the dark on MS basal medium for 24 h followed by incubation in MS basal medium containing 50 µM SA for 12 h. The explants were pre-treated with liquid MS medium with 0.1 M HCl for 1 h before visualizing at 480 nm fluorescence emission.

### Quantitative Real-time PCR (qRT-PCR) analysis of infected untransformed and BjNPR1-transgenic plants

To study the differences in the transcript levels of endogenous genes in the untransformed and *BjNPR1*-transgenic plants, seeds of T_3_ homozygous plants of ICMP451-*BjNPR1* and the untransformed plants were germinated in pots and were infected with the sporangial suspension of *S. graminicola* strain *Sg* 384 as described above. Total RNA was isolated from ICMP451-*BjNPR1*transgenics and untransformed ICMP451 using the Spectrum^TM^ plant total RNA kit (Catalog No: STRN50; Sigma). First strand cDNA was synthesized from these plants before infection, 1 day and 5 days after infection using the SuperScript III first-strand synthesis system for RT-PCR (Catalog No:18080–051; Invitrogen) and were used as template for qRT-PCR analysis. qRT-PCR analysis was carried out using the primers; 5′- AGGTGTGGAGCGGTGCGT- 3′ and 5′- TGAATGCGCTTCGAGCTATC -3′ for UDP-Salicylic acid glucosyl transferase (*SAGT*), 5′- GCTGGGTTGTAG TTGCAGATG- 3′ and 5′- GCTGGGTTGTAGTTGCAGAT G -3′ for *PR1* gene and 5′- ATGCGCTCCTGGCCTT ACT -3′ and 5′ -TCATTACTC CGATCCCGAAG- 3′ for 18s rRNA [62]. The qRT-PCR analysis was performed using Eurogentec SYBR Green qPCR Master mix with Real-Plex4 (Eppendorf, Hamburg, Germany) for 35 cycles. Later, the products were analyzed through a melt curve analysis to check the specificity of PCR amplification. Each reaction was performed twice, and the relative expression ratio was calculated using the formula, 1/2^ [Ct (18s rRNA) – Ct (gene)]^ where 2 represents perfect PCR efficiency.

### Salicylic acid tolerance test

Seeds of both the untransformed ICMP451 and the T_4_ homozygous ICMP451-*BjNPR1* transgenics were germinated on MS medium supplemented with 0, 50 µM, 100 µM, 150 µM, 200 µM, 250 µM, 300 µM and 400 µM SA and incubated in the dark at 25°C for 2 days, and at 28°C under long-day conditions for 7 days. The percentage of seed germination at each concentration was recorded.

### Statistical analysis

qRT- PCR experiments were conducted in three replications and data was analyzed using the sigma plot software, version 12.0, for windows (SPSS, Richmond, CA, USA).
